# Engineered metabarrier as shield from seismic surface waves

**DOI:** 10.1038/srep39356

**Published:** 2016-12-20

**Authors:** Antonio Palermo, Sebastian Krödel, Alessandro Marzani, Chiara Daraio

**Affiliations:** 1 Department of Civil, Chemical, Environmental and Materials Engineering, University of Bologna, Bologna, Italy; 2 Department of Mechanical and Process Engineering, Swiss Federal Institute of Technology (ETH), Zürich, Switzerland; 3 Engineering and Applied Science, California Institute of Technology, Pasadena, California 91125, USA

## Abstract

Resonant metamaterials have been proposed to reflect or redirect elastic waves at different length scales, ranging from thermal vibrations to seismic excitation. However, for seismic excitation, where energy is mostly carried by surface waves, energy reflection and redirection might lead to harming surrounding regions. Here, we propose a seismic metabarrier able to convert seismic Rayleigh waves into shear bulk waves that propagate away from the soil surface. The metabarrier is realized by burying sub-wavelength resonant structures under the soil surface. Each resonant structure consists of a cylindrical mass suspended by elastomeric springs within a concrete case and can be tuned to the resonance frequency of interest. The design allows controlling seismic waves with wavelengths from 10-to-100 m with meter-sized resonant structures. We develop an analytical model based on effective medium theory able to capture the mode conversion mechanism. The model is used to guide the design of metabarriers for varying soil conditions and validated using finite-element simulations. We investigate the shielding performance of a metabarrier in a scaled experimental model and demonstrate that surface ground motion can be reduced up to 50% in frequency regions below 10 Hz, relevant for the protection of buildings and civil infrastructures.

Protection of buildings and critical infrastructures from seismic hazards has been one of the main targets in the field of civil engineering over the last century. The first seismic design criteria aimed at achieving adequate structural resistance to withstand seismic induced loads^1^ is limiting the structural behavior to the linear elastic regime. Actual technical solutions try to exploit structural member ductility^2^ or energy damping devices^3^ to allow for a lighter structural design. Additionally, isolation devices can be used to shift the structural resonances away from the most energetic frequencies of seismic spectra (1–10 Hz). In spite of the available technical solutions for seismic resistant structures, seismic events still remain the most harmful natural hazards^4^. In fact, only a minor portion of the current building heritage has been designed according to modern seismic criteria, even in first world countries. Additionally, man induced seismic activity, possibly triggered by oil/gas exploration^5^,^6^, is becoming a major issue in regions that do not show natural seismicity and where buildings are not designed for it.

The design of structures or barriers to protect selected urbanized areas or isolated buildings from incoming seismic waves is crucial, and represents a breakthrough for the safety and for the preservation of historic and strategic infrastructures (e.g., hospitals, power plants, etc.). Recently, artificially engineered materials, designed to interact with propagating waves, have been proposed to shield large urbanized areas from seismic waves. These materials, originally introduced as seismic metamaterials^7^, are inspired by physical concepts well established in wave propagation control, like phononic crystals and acoustic metamaterials. Phononic crystals and resonant metamaterials allow to manipulate the propagation of waves at different scales, ranging from thermal^8^,^9^ to infrasound vibrations^10^,^11^,^12^,^13^,^14^. In particular, phononic crystals are periodic materials that can exhibit large and omnidirectional band gaps^15^,^16^. These band gaps are frequency regions where the propagation of waves is forbidden and arise at wavelength in the order of material periodicity. Conversely, resonant metamaterials consist of a medium with embedded locally resonant units able to interact with propagating waves at a subwavelength scale^15^,^17^.

Large scale experiments showed that phononic crystals made of cylindrical holes in sedimentary soil can reflect seismic elastic energy, achieving attenuation of ground accelerations at a frequency range around 50 Hz^7^. More recently, it has been shown that a similar concept can be used to realize seismic lenses with large (≈100 m) gradient index able to reroute surface waves around buildings^14^. However, both ideas require large structures to function at the low frequencies characteristic of seismic events (1–10 Hz).

Sub-wavelength shielding structures have been proposed to isolate buildings from incoming seismic bulk waves^18^,^19^. However, far from the epicenter bulk waves convey just a minor portion of the elastic energy released in a seismic event, while Rayleigh waves that travel along surface carry most of the energy^20^. Recently, it has been shown that Rayleigh waves interacting with array of trees can create surface wave hybridization band gaps^21^,^22^. The longitudinal resonances of trees couple with the vertical component of the Rayleigh wave and attenuate the surface ground motion by redirecting part of the elastic energy into the bulk. Surface waves deflection is advantageous for seismic wave isolation, since the elastic energy is neither reflected nor rerouted on the surface, where waves could remain harmful for surrounding regions. However, the forest metamaterial showed a working frequency range around 40 Hz, well above of the relevant seismic frequency range. Indeed, as pointed out by the authors, an engineered array of vertical pillars could be designed to reach the desired frequency target. Nonetheless, a forest of man-made pillars would result in a strongly intrusive and practically infeasible solution in a highly urbanized context. For this reason, we propose a feasible and effective seismic metabarrier in which soil-embedded surface resonators are used to redirect the surface waves into the bulk. Soil-embedded resonators, with single^23^ and or varying natural frequencies^12^, can target the required frequency range and constitute the building block of the seismic metabarrier. Here, we first develop an analytical model to guide the design of the resonant structures of the metabarrier. Next, we show using numerical simulations the effectiveness of the seismic metabarrier in attenuating surface ground motion within the 1–10 Hz range. Finally, we demonstrate Rayleigh waves attenuation on scaled experiments, which quantitatively agree with the attenuation predicted by the numerical simulations.

## Results

### Resonant metabarrier for Rayleigh waves mitigation: analytical design and numerical verification

**Figure 1:**
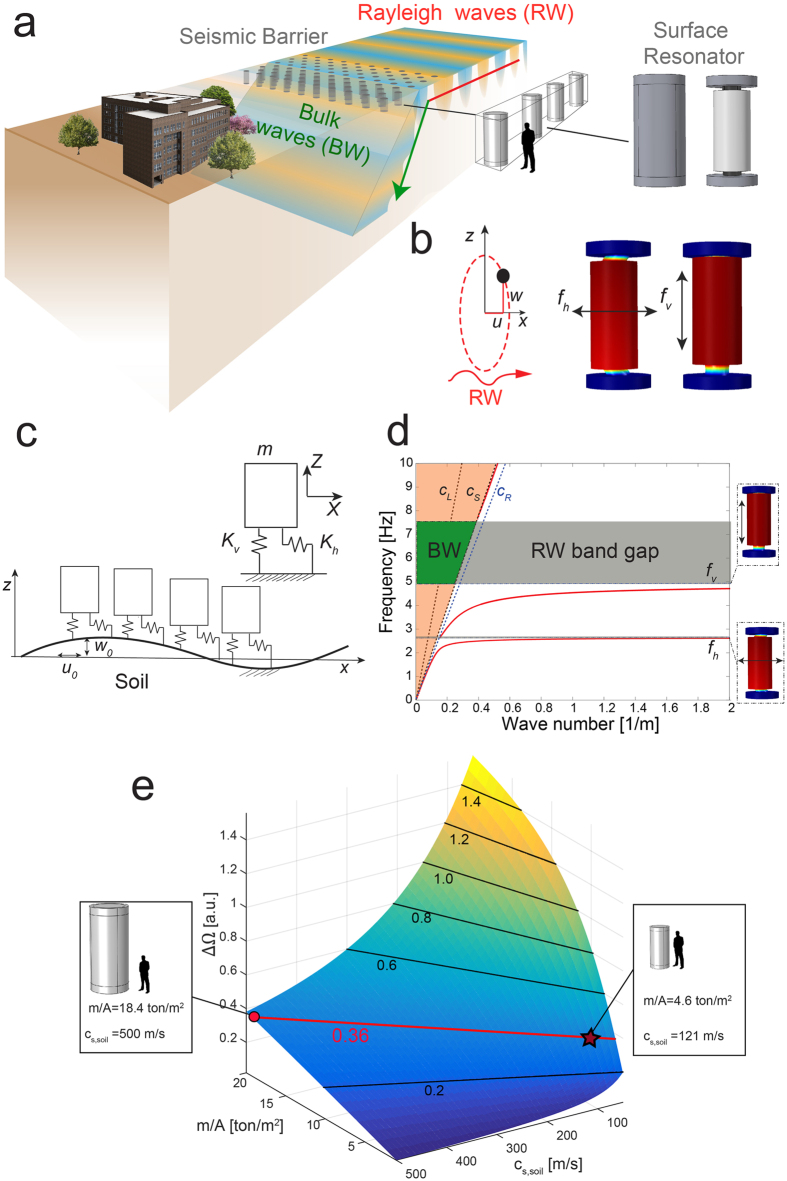
Seismic Rayleigh wave attenuation by surface resonators. (**a**) Seismic barrier of surface resonators. (**b**) Resonator modes excited by Rayleigh wave elliptical motion. (**c**) Schematic of resonators interacting with surface waves. (**d**) Dispersion relation. Geometrical and mechanical parameter of the system are collected in Table S1. Solid red line is the dispersion calculated using our model; dashed black lines are the bulk waves in the soil, while dashed blue line is the soil Rayleigh wave. The sound cone is highlighted in orange while surface band gaps are highlighted in gray. (**e**) Normalized bandwidth for varying soils vs. resonator design. Solid lines identify iso-bandwidths. The star represents the considered case, the dot represents a resonator with the same bandwidth embedded in a soil with ∼5 time higher *c*_*S,soil*_. We assume a constant soil density 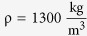 and a constant soil Poisson ratio ν_s_ = 0.3 (i.e. a constant ratio between soil longitudinal and shear wave speed 
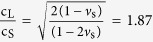
).

We study a seismic metabarrier consisting of an array of resonators buried at the soil surface^12^, around sensitive buildings (Fig. 1a). The resonant units are realized with materials commonly used in civil engineering and construction: each resonator contains a heavy cylindrical mass made of steel (Young modulus E_s_ = 210 GPa, Poisson ratio ν_s_ = 0.3, mass density ρ_s_ = 7800 kg/m^3^, with radius r_r_ and height h_r_, and an overall mass m) encased in a concrete hollow tube (E_c_ = 30 GPa, ν_c_ = 0.3, ρ_c_ = 2500 kg/m^3^, with outer radius r_c_ and height h_c_) and suspended by commercial elastic bearings (E_b_ = 1.9 MPa, ν_b_ = 0.3). An array of these resonators proved to be effective in attenuating shear and longitudinal seismic excitations exploiting their horizontal resonances^12^. Here for each resonator, we consider both the horizontal and vertical mode of vibration, with their resonant frequencies f_h_ and f_v_, respectively, as both modes are excited by the elliptical surface motion of the Rayleigh waves, as shown in Fig. 1b. Variations in the resonator mass/geometry as well as in the bearing stiffness/dimensions allow tuning the resonance frequencies within the relevant spectrum of seismic events (1–10 Hz).

The resonators are designed to be embedded in soft sedimentary soil (e.g. shear velocity c_*S*,soil_ = 100–500 m/s) and arranged in close-packed triangular lattices with a lattice constant *a*. The resonant unit dimensions and the resonators spacing *a* are subwavelength in the frequency range of interest, i.e. 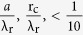, where 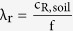 is the Rayleigh wavelength at frequency f and c_R,soil_ the Rayleigh wave velocity in the sedimentary soil.

We analyze the coupling between Rayleigh waves and such resonant structures by extending an analytical model, previously used to study the interaction of surface acoustic waves with contact resonances induced by silica microspheres adhered to a substrate^24^.

The adopted analytical model is schematically shown in Fig. 1c: the soil is represented by a semi-infinite homogeneous elastic space (*z* < 0) and each resonator by a 2-degree of freedom system (i.e. X, Z are respectively the horizontal and vertical displacements of the resonator relative to the ground surface). Each resonator has mass m = A_r_h_r_ρ_s_ and translational stiffness 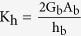 in the horizontal direction and 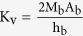 in the vertical direction, where G_b_ and M_b_ are the shear and bulk moduli of the elastic bearing material. The height h_c_ of the resonator is negligible with respect to the Rayleigh wave penetration depth d_r_, (typically d_r_ = 1.5λ_r_) allowing to treat each resonant unit as an effective boundary condition for the semi-infinite half space.

We remark that the use of plate models or further reduced 1D mass in mass-spring models could lead to incorrect conclusions on the coupling between seismic waves and resonators, as pointed out in ref. 21.

We study the dynamics of a resonator subjected to the base excitation induced by the soil surface displacements u_0_, w_0_:


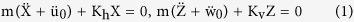


An effective medium approach is used to recover the normal and tangential stresses exchanged from soil and resonator at the surface:


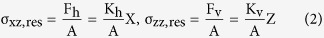


where 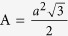 is the area over which each resonator force is considered distributed uniformly.

In doing so, we assume that the particular lattice symmetry of the resonator does not introduce directional effects on the soil-resonator dispersive properties. This assumption, that has been numerically proven for resonant pillars over silica substrate in ref. 25, holds as long as the subwavelength regime of the resonators is respected.

The analytical expression for the surface wave dispersion relation is thus obtained following a standard approach for Rayleigh waves^20^, where the stress-free boundary conditions are substituted with the normal and tangential stresses in Eq. 2 (see Supplementary Information). We seek for the real roots ω(k) of the dispersion relation (Eq. s10 Supplementary Information) assuming a resonator of mass m = 6700 kg and horizontal and vertical resonances f_h_ = 2.6 Hz and f_v_ = 4.9 Hz, respectively, embedded in a soft sedimentary soil 
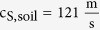
 with a triangular arrangement of lattice constant *a* = 1.3 m (see Fig. 1d). All the geometrical dimensions and mechanical parameters of the sedimentary soil and the resonators are collected in Table S1.

The Rayleigh mode hybridizes with the surface resonances and splits in three branches following a typical avoided crossing behavior^24^,^26^. Around the vertical and horizontal resonant frequencies, the solutions become strongly dispersive and terminate at the sound line, i.e. the dispersion relation of the shear bulk mode in the substrate (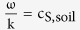). In the sound cone 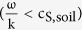 surface solutions are no more stable and become radiation modes^27^. Indeed, such radiative modes are bulk shear waves that escape from the surface^22^. As a result, the elastic energy traveling on the surface as Rayleigh waves is deviated into the bulk and the surface motion is strongly attenuated.

The mode conversion results in surface waves band gaps, i.e. frequency ranges where surface waves solutions cannot exist. For the designed resonators two surface waves band gaps emerge, each one bounded between a resonant frequency and the intersection of the upper dispersive branch and the sound line. The band gap above the horizontal frequency is extremely narrow (2.6–2.7 Hz, normalized bandwidth ΔΩ ≪ 0.05) and thus unsuitable for seismic shielding purposes. Above the vertical resonance, a wider band gap emerges (4.9–7.5 Hz, normalized bandwidth ΔΩ = 0.42), which is suitable for large spectrum seismic isolation.

To identify the relevant soil-resonator parameters driving the main band gap formation, we approximate the analytical dispersion relation by neglecting the horizontal resonance. By doing so we are able to derive a conservative close form expression for the band gap size (see Supplementary Information):


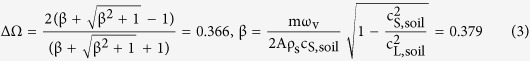


In Eq. 3, the non-dimensional parameter β can be interpreted as the ratio between the resonator mass and the mass of the soil excited by the surface wave at the vertical resonance frequency. Higher parameters β result in wider band gaps. In particular, although the resonator dimensions are designed for the assumed soft sedimentary soil, the discussed results can be generalized to harder sedimentary soils with a proper scaling of the resonator dimensions. We calculate the normalized bandwidth of resonators with different ratios of resonator mass over resonator lattice area 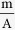, embedded in sedimentary soil with shear wave speed c_S,soil_ in the range between 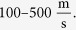 In Fig. 1e we show the normalized bandwidth for different resonators with a vertical frequency f_v_ = 4.9 Hz. The plot indicates how resonators can be designed to achieve a certain normalized bandwidth (i.e. desired attenuation capabilities) for different sedimentary soils, thus supporting the initial design of the seismic metabarrier.

**Figure 2:**
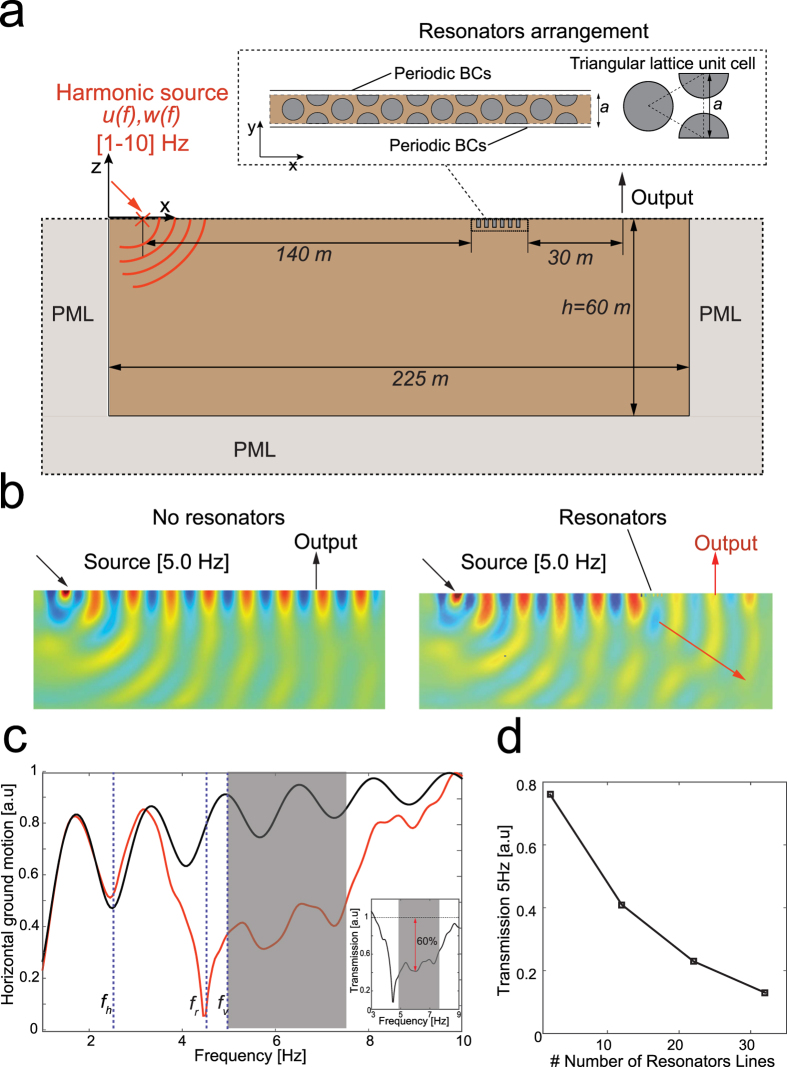
Numerical simulation of steering mechanism. (**a**) 3D stripe finite element model of soil engineered with 12 lines of resonators. (**b**) Harmonic response at 5 Hz with and without the resonators. (**c**) Horizontal surface ground motion. The output displacement at 30 m away from the resonator barrier (solid red line). The displacement is normalized with respect to the maximum of the reference field (solid black line), evaluated at the corresponding output position. We maintain constant the distance between the actuation and the measurement (output) point. As the wavelength of the surface waves varies with the excitation frequency, the measurement point can be located either in a valley or in a crest of the spatial wave profile, resulting in an apparent fluctuation of the surface displacement vs. the input wave frequency. (**d**) Horizontal ground motion attenuation as a function of the number of resonators in the array (5.0 Hz), averaged over a distance of 20 m at 30 m away from the resonator barrier.

Full-scale finite element simulations are used to confirm the analytical predictions and get further insights on the mode conversion mechanism. To this aim, we use a 3D stripe model with 12 lines of resonators in a triangular lattice arrangement (see Fig. 2a). We assume the same mechanical and geometrical parameters as in the analytical model (Table S1). The stripe has a width equal to the triangular lattice constant *a*, a length l = 225 m and a depth of h = 60 m sufficient to model the propagation of several Rayleigh wavelengths in the band gap frequency region. All materials are assumed to be linear elastic and a small isotropic loss factor (ζ_b_ = 0.05) has been used for the elastic bearing materials to avoid numerical issues at the resonators resonances. Periodic boundary conditions along *y*-direction (see inset Fig. 2a) and perfectly matched layer (PML) at the sides and at the bottom of the model avoid wave reflections from the boundaries.

We perform harmonic simulations from 1 to 10 Hz with an imposed surface displacement (u_0_(f) = w_0_(f) = d_0_e^iωt^, d_0_ = 0.01 m) applied as a line source (Fig. 2a). An identical 3D stripe model without resonators is used to obtain the reference displacement field in the analyzed frequency range. The line source is placed 140 m away from the first line of resonators and generates both bulk waves propagating in the soil as well as Rayleigh waves travelling at the surface. In the far field of the source, surface displacements are dominated by Rayleigh waves so that the interaction between resonators and bulk waves becomes negligible. The performance of the resonators is evaluated by analysing the amplitude decay of the surface ground motion after the barrier with respect to the model without barrier.

In the band gap frequency range (i.e. at 5.0 Hz), the Rayleigh waves incident to the resonator array excite the vertical resonances that in turn steer the elastic energy into the bulk, thus attenuating the surface ground motion (Fig. 2b). This phenomenon is in line with the expected surface mode conversion. For comparison, we show the reference displacement field at the same harmonic excitation (Fig. 2b). The amplitude of the horizontal ground motion u(f) for the soil-resonator system and for the reference model are evaluated 30 m away from the last line of resonators (Fig. 2c). No significant discrepancy between the reference and the soil-resonator displacement is observed at the horizontal resonant frequency f_h_, confirming the negligible effect of such resonant mode for seismic wave attenuation. On the contrary, in the second band gap region predicted analytically, the soil-resonator ground motion is strongly attenuated, with a transmission dip up to 60% at 6 Hz (see inset Fig. 2c). The attenuated frequency range is slightly larger than the analytical prediction, with a narrow transmission dip occurring before the lower band gap limit caused by the coupling between surface waves and the resonator rotational mode characterized by a resonant frequency of f_r_ = 4.6 Hz. By adding this rotational mode to the analytical model, we obtain an additional flat branch in the dispersion relation that explains the observed coupling (see Supplementary Information). All the above observations remain valid for vertical ground motion w, given the elliptical nature of the surface waves.

We use an identical numerical model with increasing number of resonators to investigate the achievable attenuation performance of the proposed seismic barrier. In Fig. 2d we show the amplitude reduction at 5 Hz for a seismic barrier with up to 32 lines of resonators. With 32 lines of resonators the surface amplitude displacement is attenuated by approximately one order of magnitude. We underline that the adopted periodic resonator arrangement is not strictly required to induce band gaps formation, as the band gap mechanism does not rely on Bragg scattering. In fact, the effectiveness of band gap formation relies on the density of resonators and not on their actual arrangement^28^. Therefore, we choose a triangular lattice that has the highest density for a given lattice constant.

### Equivalent experimental seismic metabarrier

**Figure 3:**
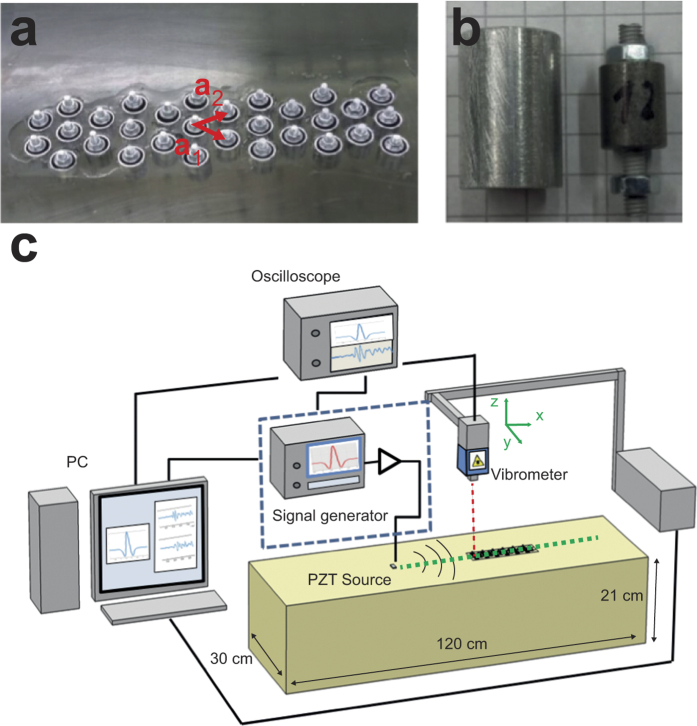
Tabletop experimental setup. (**a**) Resin block with embedded 30 resonators in a triangular lattice. (**b**) The resonator consists of rigid aluminum shell, a heavy steel mass and soft spring. (**c**) Experimental setup to measure the surface out-of-plane velocity wave field using a laser doppler vibrometer.

To experimentally verify our numerical findings, we built a tabletop experimental setup (Fig. 3a) characterized by a very similar dimensionless parameter β. For this purpose, we used polymer resin (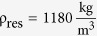, shear and longitudinal wave speeds 
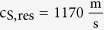
 and
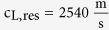
), which is commonly used in geophysical experiments^29^,^30^, to build a block (overall size of 1,2 × 0,3 × 0,21 m) representing our soil. We realized and embedded 30 small-scale resonators (see Methods) in the resin block. The resonators are placed over 12 lines in a triangular packing with a lattice constant of 1.7 cm (Fig. 3a). Each resonator consists of three main parts: a rigid aluminum tube, a soft spring and a heavy mass m_s_ ≅ 4 g (Fig. 3b). We encased the heavy steel mass in the aluminum tube. A thin, threaded aluminum rod realizes the soft spring. Only the bottom side of the rigid tube was closed allowing for a direct optical measurement of the resonator mass response during the experiments. Tuning of the resonance frequency is achieved by changing the free length of the threaded rod. In our experiments, we fix the resonance frequency to f_v,exp_ = 11.5 kHz with a measured accuracy of 10% (see Supplementary Information). This configuration results in a β_exp_ = 0.372 with an analytically predicted band gap between 11.5 kHz and 16.4 kHz (corresponding to the effective band gap width of 4.9–7.5 Hz in a real-size experiment). At the resonance frequency, the wavelength of the propagating Rayleigh waves (λ ≈ 0.1 m) is much larger than the lattice constant, in accordance with the subwavelength assumption of the model.

The out-of-plane *z* component of the surface velocity is measured using a laser doppler vibrometer mounted on a scanning stage (Fig. 3c). Rayleigh waves are excited by a piezoelectric transducer glued on the surface of the resin block 22 cm away from the resonator array (see Methods). Additionally, the same excitation setup is realized on the backside of the resin block, as control experiment to obtain the reference wave field without the influence of resonators.

**Figure 4:**
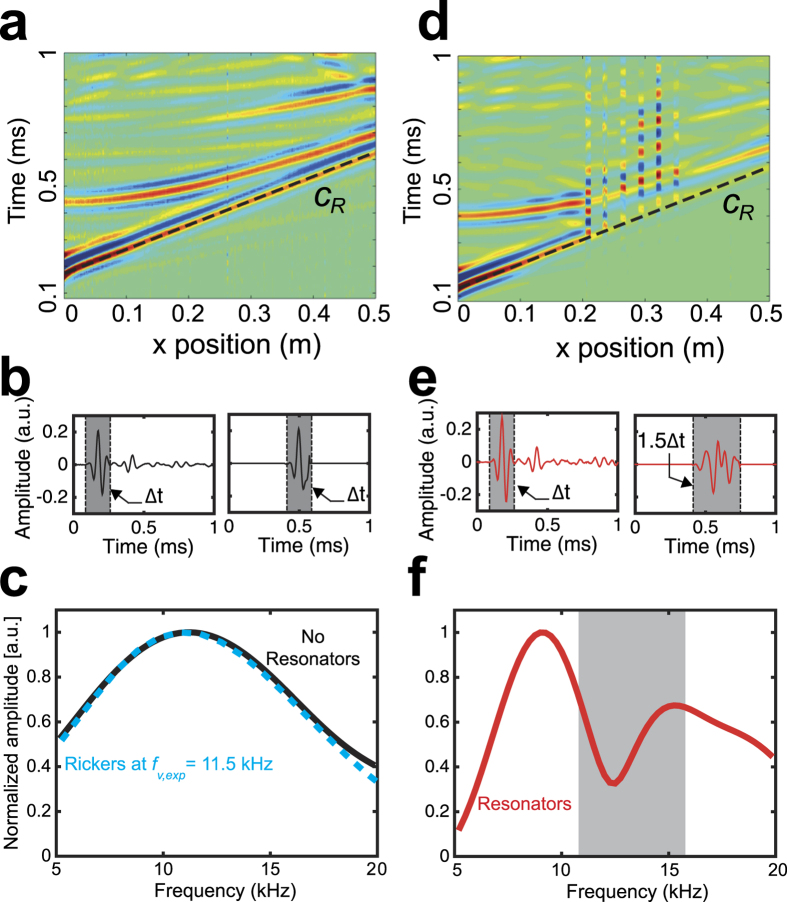
1D seismographs and frequency transmission. (**a**) Experimental seismograph of free propagation. (**b**) Transient response at 5 cm and at 35 cm distance from the source. (**c**) Normalized spectrum of the far field response compared to the spectrum of the adopted Ricker’s wavelet pulse. (**d**) Experimental seismograph of propagation with resonators. (**e**) Transient response at 5 cm and at 35 cm (after the resonators) distance from the source. (**f**) Normalized spectrum of the far field response.

We investigate the propagation of Rayleigh waves excited by a Ricker wavelet, centered at the resonance frequency f_v,exp_. Ricker wavelets are commonly used in seismology because they resemble the asymmetric frequency content of seismic waves^31^. By performing a line scan of measurements along the symmetry axis of the block, 1D seismographs are obtained from both sides of the resin block (Fig. 4a and d). The reference seismographs obtained from the resonator-free backside of the resin block is shown in Fig. 4a. We observe the non-dispersive Rayleigh wave at propagating at speed 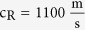 with a measured amplitude decay of 0.8 Np/m induced by material damping. Additionally, several hyperbolic wave arrivals occur, which stem from reflections of surface waves from the block’s side edges.

A moving time window Δt of duration equal to the pulse length is applied to the acquired transients in order to extract the Rayleigh wave content at the different *x* positions (Fig. 4b). We analyze the frequency content of the arriving windowed pulses at *x* = 0.38 m away from the excitation point. In the case of free propagation, the frequency spectrum matches well the theoretical spectrum of the actuated Ricker’s wavelet (Fig. 4c). The seismograph of the Ricker pulses travelling through the resonators array is depicted in Fig. 4d. As it can be seen, the surface motion excites the resonators. In turn, incident Rayleigh waves are highly attenuated after passing through the array, as predicted by the analytical model. Comparison between the initial pulse at the source (Fig. 4e, left) and the signal after the resonators (Fig. 4e, right) reveals strong attenuation and dispersion induced by the hybridization at resonance. Indeed, the frequency spectrum (Fig. 4f) shows a frequency dependent attenuation of up to 50%, centered in the predicted band gap highlighted in gray. The reached attenuation in the experimental setup is consistent with the one predicted for the real scale numerical model. The small discrepancy can be mainly attributed to transient effects due to the limited quality factor (Q ≈ 10, see Supplementary Information) of the resonators, which creates a finite activation time of the resonators.

**Figure 5:**
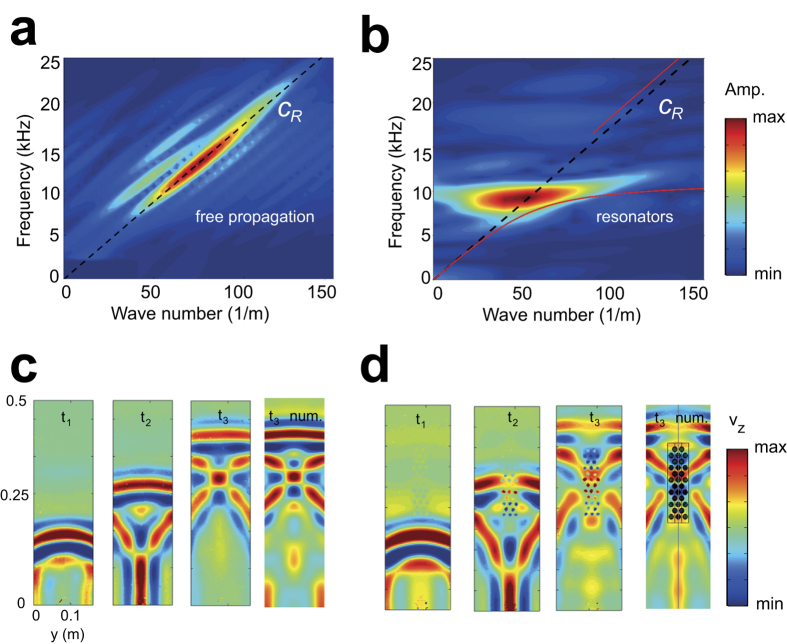
Experimental dispersion and surface velocity field. (**a**) Experimental dispersion diagram of free propagation of Rayleigh waves. (**b**) Experimental dispersion diagram of the propagation of Rayleigh through the metastructure. (**c**) Full velocity field of experimental Rayleigh wave propagation at 3 sequential time instants (t_1_ = 0.26 ms, t_2_ = 0.38 ms and t_3_ = 0.5 ms) on the free surface. Numerical comparison for time instant t_3_. (**d**) Full velocity field of experimental Rayleigh wave propagation at 3 sequential time instants (t_1_ = 0.26 ms, t_2_ = 0.38 ms and t_3_ = 0.5 ms) on the surface with the metastructure. Numerical comparison for time instant t_3_.

On the base of the 1D line measurements, we use a 2D Fourier transform to derive the experimental dispersion diagrams for the propagation of surface waves on the free surface (Fig. 5a) and along the resonators array (Fig. 5b). The propagation through the resonator array shows a highly dispersive character, as opposed to non-dispersive behavior of Rayleigh propagating on the free surface. The propagating components are spread over a broader range of wavenumbers around the resonators resonant frequency f_v,exp_ = 11.5 kHz and match well with the predicted analytical dispersion curve. The harmonic response of the resonators dominates the spectrum, and the Rayleigh solutions above the upper edge of the predicted band gap are not clearly visible.

To obtain a full field visualization of Rayleigh wave propagation, we performed 2D scans of our sample using a grid of points on the block surface (Fig. 5c,d). In the sample without resonators, (Fig. 5c), a clear plane wave front propagates, followed by wave reflections from the side boundaries of the block that generate interference figures. We performed time transient 3D finite element simulations to verify the experimental results. In the numerical simulations, we assumed a point load as a source of excitation, driven by the same Ricker wavelet as input pulse. For the free propagation (Fig. 5c), the measured experimental wave field at t3 (third panel in Fig. 5c) agrees very well with numerically simulated wave field at the same time instant (fourth panel in the same figure). In the presence of the seismic metabarrier (Fig. 5d), the wave front impinging the resonators is split and the Rayleigh energy is confined between the array and the block boundaries. Again, excellent agreement with the numerical simulations can be observed for the time instant t_3_ (compare the third and fourth panel of Fig. 5d). The Rayleigh waves passing through the resonators show large dispersion and wave attenuation. In addition, the second peak of the initial pulse is attenuated more than the first peak due to the fact that resonators are closer to steady state resonance. Far away from the array, the signal amplitude recovers due to diffraction of waves previously confined at the boundaries.

## Discussion

The attenuation bandwidth and magnitude of surface waves obtained by the resonant metabarrier depends on the interaction between the dynamic mass of soil moved by Rayleigh waves and the moving mass of the resonators. We use an analytical model to derive a unifying parameter β in Eq. (3) able to describe such interactions. This parameter allows designing seismic barriers for varying soil conditions. Specifically, we have numerically proven that in soft sedimentary soils b values of the order of 0.38 can open band gaps in the 4–7 Hz providing a reduction in the surface ground motion up to the 60%. We first verified these findings by means of harmonic finite element simulations. Next, we used the analytical model to design a small-scale equivalent experimental setup characterized by a β ≅ 0.38 and validated the numerically predicted performance of the metabarrier.

We recognize that real soils might present complex stratigraphy, leading to depth dependent wave speeds that could result in ray bending effects^32^. In such case, multi-layered models will be necessary to analyze the feasibility of the proposed seismic shielding solution. Additionally, both structural integrity and soil bearing capacity of the resonant barrier needs to be investigated for high dynamic loads induced by large magnitude events. Indeed, commercial elastomeric bearings, that are originally designed to isolate buildings and infrastructures, can certainly sustain the expected loads induced by the resonant mass exploiting either viscous or hysteretic dissipation. Whereas, for what concern soil bearing capacity the mass over area ratio provides a first indication of the required soil-strength to sustain the designed resonators, and the obtained range of values are within the bearing capacity of sedimentary soil^33^. Moreover, standard soil compaction techniques as well as appropriate foundations can be used to ensure adequate bearing capacity under dynamic excitations. Future studies will take into account the effect of resonators damping and their activation time on the effectiveness of the metabarrier. We showed that these barriers can be designed for a specific critical infrastructure, by targeting its resonance frequency. Additionally, to obtain a broadband performance of the seismic barrier the resonators frequencies could be varied along the lattice, exploiting the rainbow trapping phenomenon^12^,^22^. This could be particularly useful for shielding large urbanized areas where infrastructures and building resonances are spread over the full harmful seismic spectrum.

Integrating complex soils behavior and more sophisticated engineered resonators in the developed analytical/experimental framework will bridge the gap between the discussed metabarrier design and real scale civil engineering applications.

## Materials and Methods

### Numerical Methods

Harmonic simulations on the 3D stripe model as well as 3D time domain simulations of the resin block were carried out using the finite element based software COMSOL Multiphysics. Accuracy of the results is ensured by using convergent meshes of quadratic tetrahedral elements.

### Fabrication of experimental setup

For fabrication of the experimental setup we poured layer by layer casting resin (Resinpal 1707™) into a wooden box. We casted multiple layers of 2 cm thickness each. After each cast we waited until the material cooled down completely to avoid thermal stresses. After having released the resin block from the wooden box we applied a reflective spray (Albedo 100™) on its top and bottom surfaces that were later used for laservibrometric surface velocity measurements.

### Surface velocity measurements

For the surface velocity measurement, we mounted a laser dopplel vibrometer (Polytec OFV-534) on a scanning stage. For pulse excitation, we used a thin piezoelectric transducer (Steminc™, 20 × 15 × 1 mm, PZT-4) that was glued on the resin block by using super glue. A signal generator (Agilent 3320 A™) connected to a voltage amplifier was used to drive the piezoelectric transducer. Signals were acquired by means of an oscilloscope (Tektronix DPO3014™). At each acquisition point we averaged 32 signals to reduce the signal to noise ratio. All instruments were controlled using a Matlab© interface.

## Additional Information

**How to cite this article**: Palermo, A. *et al*. Engineered metabarrier as shield from seismic surface waves. *Sci. Rep.* **6**, 39356; doi: 10.1038/srep39356 (2016).

**Publisher's note:** Springer Nature remains neutral with regard to jurisdictional claims in published maps and institutional affiliations.

## Supplementary Material

Supplementary Information

## References

[b1] Priestley M., Calvi G. M. & Kowalsky M. J. Displacement-based seismic design of structures. Building 23, 1453–1460 (2007).

[b2] Bertero R. D. & Bertero V. V. Performance-based seismic engineering: The need for a reliable conceptual comprehensive approach. Earthq. Eng. Struct. Dyn. 31, 627–652 (2002).

[b3] Christopoulos C. & Filiatrault A. Principles of Passive Supplemental Damping and Seismic Isolation. IUSS Press 133 (2006).

[b4] Spence R. Saving lives in earthquakes: Successes and failures in seismic protection since 1960. Bull. Earthq. Eng. 5, 139–251 (2007).

[b5] Ellsworth W. L. . Increasing seismicity in the U. S. midcontinent: Implications for earthquake hazard. Lead. Edge 34, 618–626 (2015).

[b6] van Eck T., Goutbeek F., Haak H. & Dost B. Seismic hazard due to small-magnitude, shallow-source, induced earthquakes in The Netherlands. Eng. Geol. 87, 105–121 (2006).

[b7] Brûlé S., Javelaud E. H., Enoch S. & Guenneau S. Experiments on Seismic Metamaterials: Molding Surface Waves. Phys. Rev. Lett. 112, 133901 (2014). 24745420 10.1103/PhysRevLett.112.133901

[b8] Maldovan M. Sound and heat revolutions in phononics. Nature 503, 209–17 (2013). 24226887 10.1038/nature12608

[b9] Maldovan M. Phonon wave interference and thermal bandgap materials. Nat. Mater. 14, 667–674 (2015). 26099716 10.1038/nmat4308

[b10] Shi Z., Cheng Z. & Xiong C. A New Seismic Isolation Method by Using a Periodic Foundation. Earth Sp. 2586–2594 (2010).

[b11] Kim S. & Das M. P. Artificial Seismic Shadow Zone by Acoustic Metamaterials. Mod. Phys. Lett. B 27, 1–4 (2013).

[b12] Krödel S., Thomé N. & Daraio C. Wide band-gap seismic metastructures. Extrem. Mech. Lett. 4, 111–117 (2015).

[b13] Finocchio G. . Seismic metamaterials based on isochronous mechanical oscillators. Appl. Phys. Lett. 104 (2014).

[b14] Colombi A., Guenneau S., Roux P. & Richard C. Transformation seismology: composite soil lenses for steering surface elastic Rayleigh waves. Nat. Publ. Gr. 9, 1–19 (2015). 10.1038/srep25320 PMC4850458 27125237

[b15] Deymier P. Acoustic metamaterials and phononic crystals (2013).

[b16] Hussein M. I., Leamy M. J. & Ruzzene M. Dynamics of Phononic Materials and Structures: Historical Origins, Recent Progress, and Future Outlook. Appl. Mech. Rev. 66, 40802 (2014).

[b17] Kadic M., Bückmann T., Schittny R. & Wegener M. Metamaterials beyond electromagnetism. Rep. Prog. Phys. 76, 126501 (2013). 24190877 10.1088/0034-4885/76/12/126501

[b18] Shi Z., Cheng Z. & Xiang H. Seismic isolation foundations with effective attenuation zones. Soil Dyn. Earthq. Eng. 57, 143–151 (2014).

[b19] Cacciola P. & Tombari A. Vibrating barrier : a novel device for the passive control of structures under ground motion Subject Areas: Proc. R. Soc. A Math. Phys. Eng. Sci. 471 (2015). 10.1098/rspa.2015.0075 PMC4528655 26345731

[b20] Graff K. F. Wave motion in elastic solids. Wave motion in elastic solids (1991).

[b21] Colombi A., Roux P., Guenneau S., Gueguen P. & Craster R. V. Forests as a natural seismic metamaterial: Rayleigh wave bandgaps induced by local resonances. Nat. Sci. Reports 1–7, doi: 10.1038/srep19238 (2015). PMC4707539 26750489

[b22] Colombi A., Colquitt D., Roux P., Guenneau S. & Craster R. V. A seismic metamaterial: The resonant metawedge. Sci. Rep. 6, 27717 (2016). 27283587 10.1038/srep27717 PMC4901369

[b23] Dertimanis V. K., Antoniadis I. A. & Chatzi E. N. Feasibility Analysis on the Attenuation of Strong Ground Motions Using Finite Periodic Lattices of Mass-in-Mass Barriers. J. Eng. Mech. 1–10, doi: 10.1061/(ASCE)EM.1943-7889.0001120 (2016).

[b24] Boechler N. . Interaction of a contact resonance of microspheres with surface acoustic waves. Phys. Rev. Lett. 111 (2013). 10.1103/PhysRevLett.111.036103 23909341

[b25] Khelif A., Achaoui Y. & Aoubiza B. In Acoustic Metamaterials 43–59 (Springer, 2013).

[b26] Christensen M. . Avoided crossing of rattler modes in thermoelectric materials. Nat. Mater. 7 (2008). 10.1038/nmat2273 18758454

[b27] Benchabane S., Khelif a., Rauch J. Y., Robert L. & Laude V. Evidence for complete surface wave band gap in a piezoelectric phononic crystal. Phys. Rev. E - Stat. Nonlinear, Soft Matter Phys. 73, 1–4 (2006). 10.1103/PhysRevE.73.065601 16906904

[b28] Liu Z. . Locally Resonant Sonic Materials. Science 289, 1734–1736 (2000). 10976063 10.1126/science.289.5485.1734

[b29] Bretaudeau F., Brossier R., Leparoux D., Abraham O. & Virieux J. 2D elastic full-waveform imaging of the near-surface: Application to synthetic and physical modelling data sets. Near Surf. Geophys. 11, 307–316 (2013).

[b30] Bretaudeau F., Leparoux D., Durand O. & Abraham O. Small-scale modeling of onshore seismic experiment: A tool to validate numerical modeling and seismic imaging methods. Geophysics 76, T101–T112 (2011).

[b31] Ricker N. The form and laws of propagation of seismic wavelets. Geophysics 18, 10–40 (1953).

[b32] Pavlis G. L. ‘An Introduction to Seismology, Earthquakes, and Earth Structure’ by Seth Stein and Michael Wysession. Seismological Research Letters 74 (2003).

[b33] Handy R. & Spangler M. Geotechnical engineering: soil and foundation principles and practice. (McGraw Hill Professional, 2007).

