# Supplementary material for: Engineered metabarrier as shield from seismic surface waves
Source: Sci Rep. 2016 Dec 20;6:39356. doi: 10.1038/srep39356 (PMC5172158; doi:10.1038/srep39356)
Supplement: Supplementary Information [file srep39356-s1.pdf]

Supplementary Information:

Engineered metabarrier as shield from seismic surface waves

Antonio Palermo[1,2†*], Sebastian Krödel[2*], Alessandro Marzani[1], Chiara Daraio[3]

[1]*Department of Civil, Chemical, Environmental and Materials Engineering, University of Bologna, Bologna, Italy*

[2]*Department of Mechanical and Process Engineering, Swiss Federal Institute of Technology (ETH), Zürich, Switzerland*

[3]*Engineering and Applied Science, California Institute of Technology, Pasadena, California 91125, USA*

S1. DERIVATION OF THE DISPERSION RELATION FOR RAYILEGH WAVES COUPLED WITH SURFACE THREE DEGREE OF FREEDOM RESONATORS

Elastic waves travelling in the soil substrate can by described by the wave equations $\nabla^2\Phi = \frac{1}{c_L^2}\frac{\partial^2\Phi}{\partial t^2}$, $\nabla^2 H_y = \frac{1}{c_S^2}\frac{\partial^2 H_y}{\partial t^2}$ where t is time, Φ and H_y are the dilatational and transverse potentials that for a semi-infinite elastic space take the form [1]:

$$\Phi = Ae^{kz\sqrt{1-\frac{\omega^2}{k^2c_L^2}}}e^{i(\omega t-kx)},\ H_y = Be^{kz\sqrt{1-\frac{\omega^2}{k^2c_S^2}}}e^{i(\omega t-kx)} \quad \text{(s.1)}$$

The vertical and horizontal displacement components u and w of the wave field relate to the potentials Φ and $\mathrm{H_y}$ as follow:

$$u = \frac{\partial\Phi}{\partial x} - \frac{\partial H_y}{\partial z}, \qquad w = \frac{\partial\Phi}{\partial z} + \frac{\partial H_y}{\partial x} \quad \text{(s.2)}$$

We study the dynamic of a three degree of freedom resonator subjected to the base excitation induced by the horizontal and vertical displacements $u_0 = u(z=0)$ and $w_0 = w(z=0)$, respectively, generated by a surface Rayleigh wave (Fig S1(a) and S1(b)). The resonator has three-degree-of-freedom namely the horizontal and vertical displacements of its mass with respect to the base of the resonator, X and Z, respectively, and θ the angle of rotation of the mass with respect to the z axis. The translational mass is denoted by m whereas the rotational inertia of the

mass as I. We assume that the vertical motion is uncoupled from both horizontal and rotational displacements, while the horizontal and rotational motions are coupled when the bearings are not symmetric, i.e. $\alpha = \frac{K_{h,1}}{K_{h,2}} \neq 1$, with $K_{h,i} = \frac{GA_{b,i}}{h_{b,i}}$ being the horizontal stiffness of the bottom and top elastic bearing, $K_{h,1}$, $K_{h,2}$ respectively (see Fig. S1a)

Therefore, the equations of motion of the resonator subjected to the base displacements u_0 and w_0 read:

$$m(\ddot{Z} + \ddot{w}_0) + K_v Z = 0, \tag{s.3a}$$

$$\begin{bmatrix} m & 0 \\ 0 & I \end{bmatrix}\left(\begin{bmatrix} \ddot{X} \\ \ddot{\theta} \end{bmatrix} + \ddot{u}_0 \begin{bmatrix} 1 \\ 0 \end{bmatrix}\right) + \begin{bmatrix} K_h & K_{hr} \\ K_{rh} & K_r \end{bmatrix}\begin{bmatrix} X \\ \theta \end{bmatrix} = \begin{bmatrix} 0 \\ 0 \end{bmatrix} \tag{s.3b}$$

where K_v is the stiffness along the vertical direction; $K_h = K_{h,1} + K_{h,2} = K_{h,2}(1 + \alpha)$ is the total stiffness along the horizontal direction; $K_r = \frac{K_{h,2}\alpha h_r^2}{(1+\alpha)}$ is the rotational stiffness and $K_{hr} = K_{rh} = \frac{K_{h,2}(1-\alpha)h_r}{2}$ the coupled rotational-horizontal stiffness.

For the vertical motion, we assume a wave solution of the form $Z = Z_0 e^{i(\omega t - kx)}$ and substitute it into Eq. (s.3a) to obtain [2]:

$$Z_0 = \frac{\omega^2}{{\omega_v}^2 - \omega^2} w_0 \tag{s.4}$$

where ${\omega_v}^2 = \frac{K_v}{m}$. Similarly, the horizontal-rotational Eqs. (s.3b) can be uncoupled by means of modal analysis, as:

$$\ddot{\eta}_i + {\omega_i}^2 \eta_i = \Gamma_i \ddot{u}_0 \quad \text{and} \quad \Gamma_i = -\frac{\begin{bmatrix} V_{i1} \\ V_{i2} \end{bmatrix}^T \begin{bmatrix} m & 0 \\ 0 & I \end{bmatrix}\begin{bmatrix} 1 \\ 0 \end{bmatrix}}{\begin{bmatrix} V_{i1} \\ V_{i2} \end{bmatrix}^T \begin{bmatrix} m & 0 \\ 0 & I \end{bmatrix}\begin{bmatrix} V_{i1} \\ V_{i2} \end{bmatrix}} \quad i = 1,2 \tag{s.5}$$

where ω_i is the natural frequency, V_i the eigenmode, η_i the modal displacement and Γ_i the modal participation factor. By assuming a wave solution $\eta_i = \eta_{i,0} e^{i(\omega t - kx)}$ for the resonator mode, and substituting it into Eq.(s.5) we obtain:

$$\eta_{i,0}(\omega, u_0) = -\frac{\omega^2}{{\omega_i}^2 - \omega^2} \Gamma_i u_0 \tag{s.6}$$

The horizontal and rotational displacements are thus obtained as:

$$\begin{bmatrix} X_0(\omega, u_0) \\ \theta_0(\omega, u_0) \end{bmatrix} = \sum_{i=1}^{2} \eta_{i,0}(\omega, u_0)\, V_i \tag{s.7}$$

By substituting Eqs. (s.4), (s.7) into Eq. (2) of the main text, we obtain the stress boundary conditions in terms of surface displacements u_0, w_0:

$$\sigma_{xz,res} = \frac{F_h}{A} = \frac{K_h}{A} X_0(\omega, u_0), \qquad \sigma_{zz,res} = \frac{F_v}{A} = \frac{K_v}{A} \frac{\omega^2}{{\omega_v}^2 - \omega^2} w_0 \tag{s.8}$$

that for the case considered in the main text of resonators with only 2 dofs, reduce to:

$$\sigma_{xz,res} = \frac{F_h}{A} = \frac{K_h}{A} \frac{\omega^2}{{\omega_h}^2 - \omega^2} u_0, \qquad \sigma_{zz,res} = \frac{F_v}{A} = \frac{K_v}{A} \frac{\omega^2}{{\omega_v}^2 - \omega^2} w_0 \tag{s.9}$$

A this stage, using Eq. (s1), Eq. (s9), and isotropic linear elastic stress-strain relations[1] we can obtain the dispersion relation for surface Rayleigh waves coupled with the 3-dof (and similarly 2-dof) resonators. In particular, for the case of 2-dof the dispersion relation has the following closed analytical form:

$$\begin{aligned}
&\left(2 - \frac{\omega^2}{k^2 c_{S,soil}^2}\right)^2 - 4\sqrt{1 - \frac{\omega^2}{k^2 c_{L,soil}^2}}\sqrt{1 - \frac{\omega^2}{k^2 c_{S,soil}^2}} \\
&+ \frac{\omega^4\, m}{A c_{S,soil}^4 \rho_{soil} k^3} \frac{\omega_v^2}{\omega_v^2 - \omega^2} \sqrt{1 - \frac{\omega^2}{k^2 c_{L,soil}^2}} + \frac{\omega^4\, m}{A c_{S,soil}^4 \rho_{soil} k^3} \frac{\omega_h^2}{\omega_h^2 - \omega^2} \sqrt{1 - \frac{\omega^2}{k^2 c_{S,soil}^2}} \\
&+ \frac{\omega^4\, m}{A c_{S,soil}^2 \rho_{soil} k} \frac{\omega_v^2}{\omega_v^2 - \omega^2} \frac{m}{A c_{S,soil}^2 \rho_{soil} k} \frac{\omega_h^2}{\omega_h^2 - \omega^2} \left(\sqrt{1 - \frac{\omega^2}{k^2 c_{L,soil}^2}}\sqrt{1 - \frac{\omega^2}{k^2 c_{S,soil}^2}} - 1\right) \\
&= 0
\end{aligned} \tag{s.10}$$

with $\omega_h = \sqrt{\frac{K_h}{m}}$ and $\omega_v = \sqrt{\frac{K_v}{m}}$ denoting the horizontal and vertical angular frequencies of the resonator. In Eq. (s.10) one can recognize the standard Rayleigh dispersion term (first line of Eq. (s.10)), the terms related to the vertical and horizontal resonances (second line of Eq. (s.10)) and a further term in which the horizontal and vertical resonances are coupled (third line of Eq. (s.10)). This coupling arises from the elliptical motion of surface waves.

In Fig. S1(c) we show the real roots of the dispersive relation $\omega(k)$ for the soil coupled with the 3 dof resonators (the geometrical and mechanical parameters in Table S1 of the main text have

been used). For this symmetric resonator, the two resonant modes are uncoupled and correspond to a pure horizontal translation and a pure rotation of the resonator with eigenfrequencies:

$$f_h = \frac{1}{2\pi}\sqrt{\frac{K_h}{m}} = 2.6\ Hz \quad f_r = \frac{1}{2\pi}\sqrt{\frac{K_r}{I}} = 4.6\ Hz \tag{s.11}$$

However, in a full uncoupled system the rotational motion cannot be excited by the horizontal base displacement. On the contrary in the real system, the variation of the surface wave horizontal displacement along the resonator depth induces resonator rotation. To account for this we introduce a small numerical asymmetry $\alpha = 1.01$. As shown in Fig. S1(c) the rotational motion does not substantially effect the position and size of the occurring band gap. However, we observe a further flat branch at the rotational resonance frequency.

We underline that the use of the modal analysis to extract the response of the resonator can be extended to multi-dof (or even multi-mass) resonator systems.

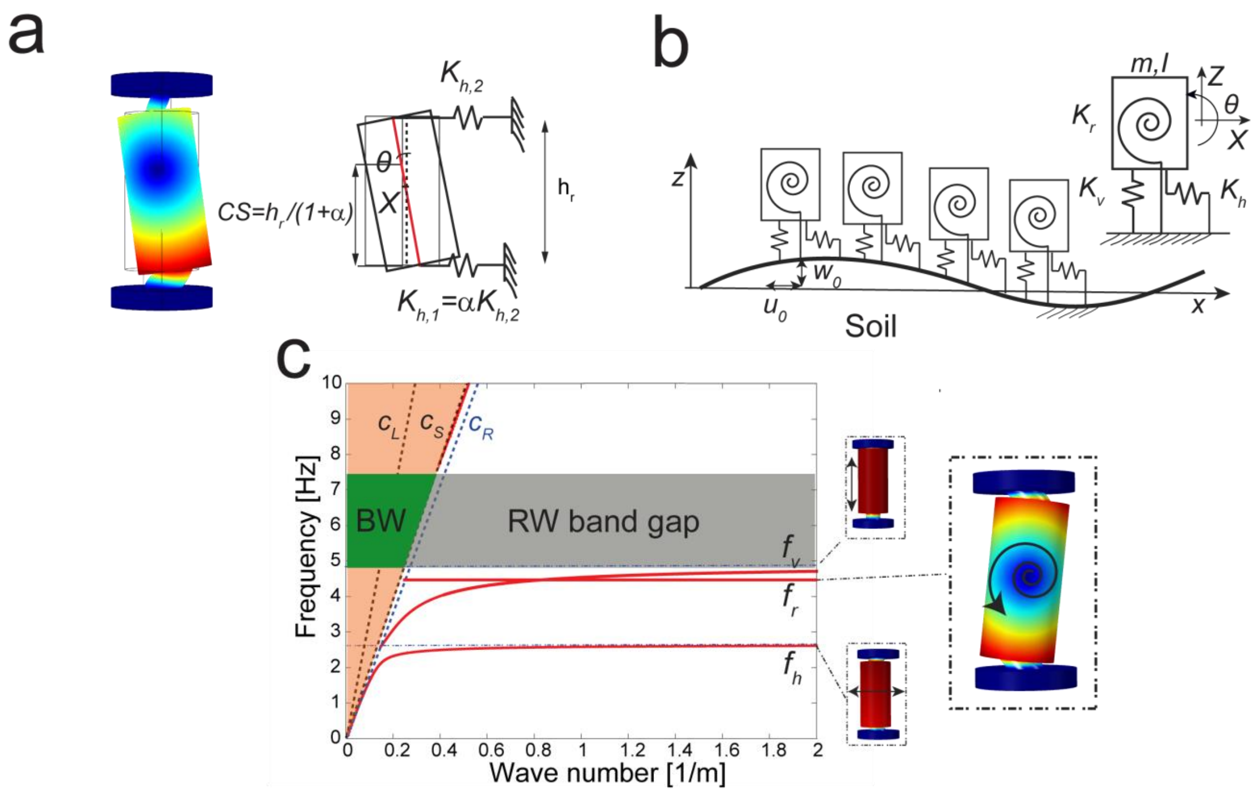

Fig. S1. Interaction of surface waves with 3-dof resonators. (a) Horizontal and rotational kinematics of the resonator. (b) Schematic of 3-dof resonators interacting with surface waves. (c) Corresponding dispersion relation.

S2 BAND GAP FREQUENCY EDGES AND NORMALIZED BANDWITH

The approximate dispersion relation for the soil-resonator system obtained by neglecting the horizontal and rotational resonances reads (2):

$$\left(\frac{\omega^2}{\omega_v^2}-1\right)\left(\left(2-\frac{\omega^2}{k^2c_S^2}\right)^2-4\sqrt{1-\frac{\omega^2}{k^2c_L^2}}\sqrt{1-\frac{\omega^2}{k^2c_S^2}}\right)=\frac{m\omega^4}{Ac_S^4\rho k^3}\sqrt{1-\frac{\omega^2}{k^2c_L^2}} \qquad \text{(s.12)}$$

Inspection of the analytical relation allows to identify the frequency of the band gap (BG) lower ω^- and upper ω^+ edges:

BG Lower edge:

$$\text{for } k \longrightarrow \infty \qquad \left(\frac{\omega^2}{\omega_v^2}-1\right)=0 \qquad \omega^-=\omega_v \qquad \text{(s.13)}$$

BG upper edge:

$$\text{for } \omega = kc_S \qquad \left(\frac{\omega^2}{\omega_v^2}-1\right)=\frac{m\omega}{A\rho c_S}\sqrt{1-\frac{c_S^2}{c_L^2}} \qquad \text{(s.14)}$$

introducing $\beta=\frac{m\omega_v}{2A\rho c_S}\sqrt{1-\frac{c_S^2}{c_L^2}}$ $\qquad \omega^+=\omega_v\left(\beta+\sqrt{\beta^2+1}\right)$

From the BG lower and upper edges the band gap normalized width

$\Delta\Omega=\frac{\omega^+-\omega^-}{0.5*(\omega^++\omega^-)}$ (Eq. (4) in the main text) is obtained.

S2. Experimental characterization of the resonator

In Fig. S2(a) we show a typical transient response of the resonator to incoming surface waves. The resonator shows a narrow spectrum centered around its resonance frequency $f_{v,exp}$ (Fig S2(b)). We found a small variation in resonance frequency and quality factor over all resonators (approximately 10%).

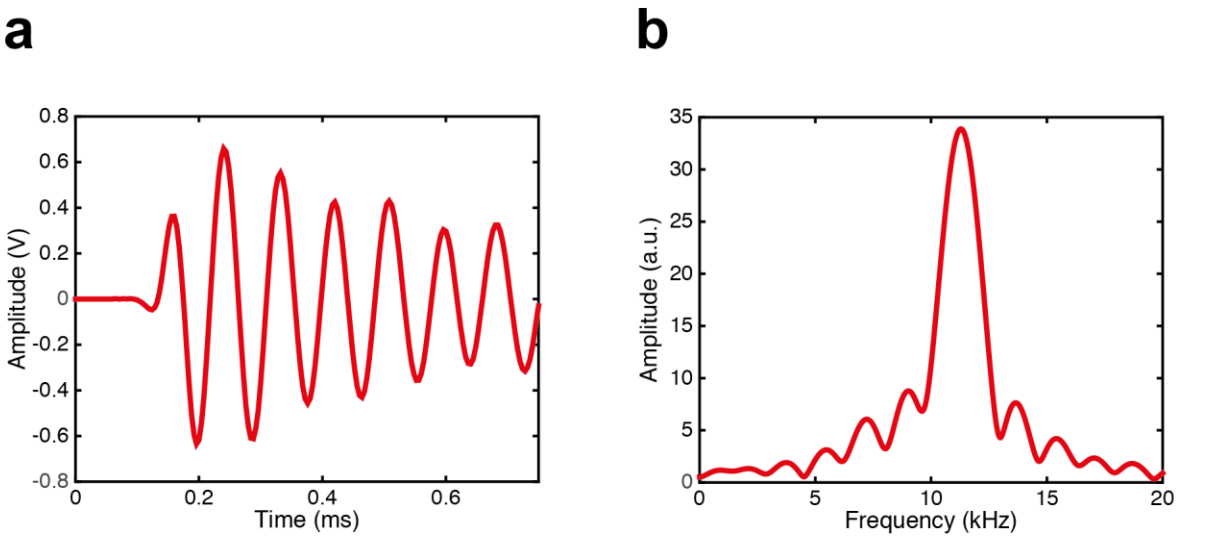

Fig. S2. Resonator response**.** (a) Typical transient response of the scaled resonator and (b) the corresponding frequency spectrum

Table

			Mechanical parameters		
	**Dimensions**		**Mechanical parameters**		
**Soil**			$E_{soil} = 50\ MPa$ $c_{L,soil} = 227\ m/s$	$\nu_{soil} = 0.3$ $c_{S,soil} = 121\ m/s$	$\rho_{soil} = 1300\frac{m}{s}$
**Resonator**	Steel mass	$r_r = 0.4\ m$ $h_r = 1.7\ m$	$E_s = 210\ GPa$	$\nu_s = 0.3$	$\rho_s = 7800\ kg/m^3$
	Elastic bearings	$r_b = 0.2\ m$ $h_b = 0.1\ m$	$E_b = 1.9\ MPa$	$\nu_b = 0.3$	$\rho_b = 1100\ kg/m^3$
	Concrete shell	$r_c = 0.55\ m$ $t_c = 0.05$ $h_c = 2.5\ m$	$E_c = 30\ GPa$	$\nu_c = 0.3$	$\rho_c = 2500\ kg/m^3$

Table S1 | Dimensions and mechanical parameters of sedimentary soil and resonators

REFERENCES

1. Graff, K. F. *Wave motion in elastic solids*. *Wave motion in elastic solids* (1991).
2. Boechler, N. *et al.* Interaction of a contact resonance of microspheres with surface acoustic waves. *Phys. Rev. Lett.* **111,** (2013).
